# Voxel-Based Analysis of Fractional Anisotropy in Post-Stroke Apathy

**DOI:** 10.1371/journal.pone.0116168

**Published:** 2015-01-02

**Authors:** Song-ran Yang, Xin-yuan Shang, Jun Tao, Jian-yang Liu, Ping Hua

**Affiliations:** 1 Department of Experimental Psychology, University of Oxford, Oxford, United Kingdom; 2 Department of Neurology, Guangzhou First People’s Hospital, Guangzhou Medical University, Guangzhou, China; 3 Department of Cardiovascular Surgery, Sun Yat-sen Memorial Hospital, Sun Yat-sen University, Guangzhou, China; University of New Mexico, United States of America

## Abstract

**Objective:**

To explore the structural basis of post-stroke apathy by using voxel-based analysis (VBA) of fractional anisotropy (FA) maps.

**Methods:**

We enrolled 54 consecutive patients with ischemic stroke during convalescence, and divided them into apathy (n = 31) and non-apathy (n = 23) groups. We obtained magnetic resonance images of their brains, including T1, T2 and DTI sequences. Age, sex, education level, Hamilton Depression Scale (HAMD) scores, Mini-Mental State Examination (MMSE) scores, National Institutes of Health Stroke Scale (NIHSS) scores, and infarct locations for the two groups were compared. Finally, to investigate the structural basis of post-stroke apathy, VBA of FA maps was performed in which we included the variables that a univariate analysis determined had *P*-values less than 0.20 as covariates.

**Results:**

HAMD (*P* = 0.01) and MMSE (*P*<0.01) scores differed significantly between the apathy and non-apathy groups. After controlling for age, education level, HAMD scores, and MMSE scores, significant FA reduction was detected in four clusters with peak voxels at the genu of the corpus callosum (X = −16, Y = 30, Z = 8), left anterior corona radiata (−22, 30, 10), splenium of the corpus callosum (−24, −56, 18), and right inferior frontal gyrus white matter (52, 24, 18), after family-wise error correction for multiple comparisons.

**Conclusions:**

Post-stroke apathy is related to depression and cognitive decline. Damage to the genu of the corpus callosum, left anterior corona radiata, splenium of the corpus callosum, and white matter in the right inferior frontal gyrus may lead to apathy after ischemic stroke.

## Introduction

Apathy is a common symptom after ischemic stroke, and is defined as “a symptom without initiative action, but which cannot be completely explained by using consciousness, depression, and cognitive dysfunction” [1]. Apathy has been reported to have a prevalence of 15.2%–71.0% in the acute phase after a stroke [2], and its occurrence can lead to reduced efficiency of stroke treatment, worse prognosis, and decline in activities of daily living [3]. The structural basis of post-stroke apathy is complicated, and studies have suggested that it is associated with damage to the frontal lobe [4], basal ganglia [5], temporal lobe [6], and other brain regions. Whereas some reports suggest that it is linked to disconnection of frontal subcortical loops [4], [5], this is still debatable given that a recent meta-analysis reported that apathy was not associated with any specific lesion location [7].

Diffusion tensor imaging (DTI) is a noninvasive technique used for evaluating the structural integrity of white matter in the brain. This is accomplished in part through measuring the fractional anisotropy (FA) which when low indicates damage or degeneration in white matter. In addition to primary lesions, this technique may provide information regarding distal fiber degeneration secondary to stroke (i.e. Wallerian degeneration) and other types of changes [8] or lesions [9] in white matter that have been linked to apathetic behavior. Additionally, the development of voxel-based analysis (VBA) [10] has made it possible to put the individual FA images into the same standard space and statistically compare whole-brain FA values voxel by voxel.

Here, we hypothesized that disconnection of certain brain circuits, especially the frontal subcortical loops, serves as a risk factor for post-stroke apathy. To test this, we used VBA to explore changes in white matter connectivity. Given that low FA is regarded as a sign of that white matter organization has degraded [8], we expected to find lower FA values in the white matter of frontal regions of patients with post-stroke apathy, especially those regions that connect frontal cortex to subcortical regions. Indeed, this turned out to be the case.

## Materials and Methods

### Patients

We enrolled consecutive patients with ischemic stroke from July 2012 to February 2013. Patients met the following inclusion criteria: age 50–80 years; diagnosed with ischemic stroke according to the International Classification of Disease 10th Revision (ICD-10) [11] criteria; cerebral hemorrhage excluded by computed tomography; 3.0 T magnetic resonance imaging (MRI) was performed within 7 days after stroke using T1, T2, and DTI sequences; cooperation during the inspection and scales assessment. Patients were excluded if they had a history of psychiatric illness (such as schizophrenia, depression or anxiety), a history of neurodegenerative disease (such as dementia or Parkinson’s disease), a history of heavy drinking or drug use, or if the quality of their MR images was poor. The study was approved by the Institutional Review Board of Sun Yat-sen Memorial Hospital (No. 024, 2012). All the patients had the capacity to consent and written informed consent was obtained from all patients before the study.

### Assessment and diagnosis of apathy

Fifty-four patients were included in the study, and scores from the National Institutes of Health Stroke Scale (NIHSS) were recorded on admission. The clinical diagnosis of apathy was based on that proposed by Robert et al [12], and the Apathy Evaluation Scale – Clinical Version (AES-C) [13] was used for auxiliary assessment. Apathy assessments and diagnoses were done within 30–45 days of stroke for all patients. According to the results of clinical diagnoses, patients were divided into two groups: 31 in the apathy group and 23 in the non-apathy group.

Assessment of depression and cognitive dysfunction were carried out using the Hamilton Depression Scale (HAMD) and Mini-Mental State Examination (MMSE).

### Imaging data and processing

MR images were obtained using a Siemens 3.0 T MRI system (Siemens Verio, Erlangen, Germany), with a standard head coil. T1 and T2 images were taken with a self-spin echo sequence, and DTI images were taken with a planar echo sequence. The DTI scan consisted of 21 diffusion-weighted directions with a b-value of 1000 s/mm^2^ and one volume without diffusion weighting (i.e., b0 image). The parameters of the DTI sequence were as follows: slice thickness = 3 mm, echo time = 86 ms, repetition time = 8300 ms, b = 1000 s/mm^2^, field of view = 240 mm×240 mm, acquisition matrix = 128×128, and in-plane resolution = 2.2.×2.2 mm.

The DTI data were preprocessed by PANDA software [14], following these steps: skull removal, correction of eddy-current distortion, and construction of FA maps. The FA maps generated for each patient were then transformed from individual space to a standard Montreal Neurological Institute (MNI) space via spatial normalization, and resliced with a voxel size of 2 mm×2 mm×2 mm. All FA maps were smoothed using an isotropic Gaussian filter with a full-width-at-half-maximum of 6 mm.

### Sites of infarction

Each brain was divided into 16 regions (bilateral frontal, parietal, temporal, and occipital lobes, and bilateral basal ganglia, thalamus, brain stem, and cerebellum), and the presence of infarction was determined through MRI T2 imaging.

### Statistical analysis

Univariate analysis of clinical data was performed with an independent sample *t-* test (two-sided) and a χ^2^ test. Data from each of the 16 different brain regions were compared between patients with and without apathy using a χ^2^ test. Statistical analysis was performed with SPSS version 15.0 (SPSS, Chicago, IL, USA). Comparison of FA values between groups was done using Statistical Parametric Mapping 8 (SPM8, Wellcome Department of Cognitive Neurology, London, UK). The testing principle was as follows: After spatial standardization and smoothing, FA values stored in each voxel at a given MNI coordinate were compared between groups using a one-sided larger than those in the apathy group. To control for confounding factors, we included the clinical variables that the univariate analysis indicated might be somewhat different between groups (*P*<0.20) as covariates. Because FA reduction also reveals information about stroke lesion, variables for lesion location were not included as covariates [15]. To avoid false-positives, we corrected for multiple comparisons using the family-wise error (FWE) method, and the statistical criteria were set at *P*<0.05 with a cluster size>50.

## Results

Of the 54 patients, 31 (57.4%) were clinically diagnosed with apathy. We found that HAMD scores (*P* = 0.01) and MMSE scores (*P*<0.01) significantly differed between patients with and without apathy, whereas age (*P* = 0.12) and education level (*P* = 0.06) also tended to differ across groups. These four variables (*P-*values <0.20) were included in the VBA as covariates. Sex and NIHSS scores did not differ significantly between the two groups, and significance levels did not exceed our 0.20 threshold (Table 1).

**Table 1:** Comparison of clinical data between groups.

Groups	Non-apathy group (*n* = 23)	Apathy group (*n* = 31)	*P* value
Age (years)^1^	65.26±8.16	68.94±8.57	0.12
Gender (Male/Female)^2^	14/9	21/10	0.60
Education level^1^	9.96±4.71	7.60±4.02	0.06
NIHSS^1^	1.22±1.41	1.61±1.91	0.41
HAMD score^1^	6.52±3.40	9.74±4.93	0.01
MMSE score^1^	27.26±2.96	23.94±4.46	<0.01
AES-C score^1^	36.87±3.58	48.52±5.05	<0.01

1 *t*-test of independent sample,

2 *χ* ^2^ test.

### Relationship between stroke lesions and post-stroke apathy

Stroke lesions were detected in the left frontal lobe in 16 patients, right frontal lobe in 18, left parietal lobe in 4, right parietal lobe in 6, left temporal lobe in 3, right temporal lobe in 3, left occipital lobe in 3, right occipital lobe in 2, left basal ganglia in 21, right basal ganglia in 18, left thalamus in 11, right thalamus in 12, left brain stem in 13, right brain stem in 8, left cerebellum in 3 and right cerebellum in 2. None of the 16 brain regions exhibited significant differences in the number of lesions between apathy and non-apathy groups (Table 2).

**Table 2:** Comparison of the number of cases with infarction between the two groups.

Infarcted region	Non-apathy group (yes/no)	Apathy group (yes/no)	*P* values
Left frontal	4/19	12/19	0.09
Right frontal lobe	6/17	12/19	0.39
Left parietal lobe	1/22	3/28	0.83
Right parietal lobe	1/22	5/26	0.36
Left temporal lobe	0/23	3/28	0.35
Right temporal lobe	0/23	3/28	0.35
Left occipital lobe	1/22	2/29	1.00
Right occipital lobe	1/22	1/30	1.00
Left basal ganglia	6/17	15/16	0.10
Right basal ganglia	5/18	13/18	0.12
Left thalamus	3/20	8/23	0.32
Right thalamus	6/17	6/25	0.56
Left brain stem	4/19	9/22	0.32
Right brain stem	2/21	6/25	0.48
Left cerebellum	1/22	2/29	1.00
Right cerebellum	1/22	1/30	1.00

All data analyzed with *χ^2^* test.

### VBA of FA maps in post-stroke apathy

**Figure 1:**
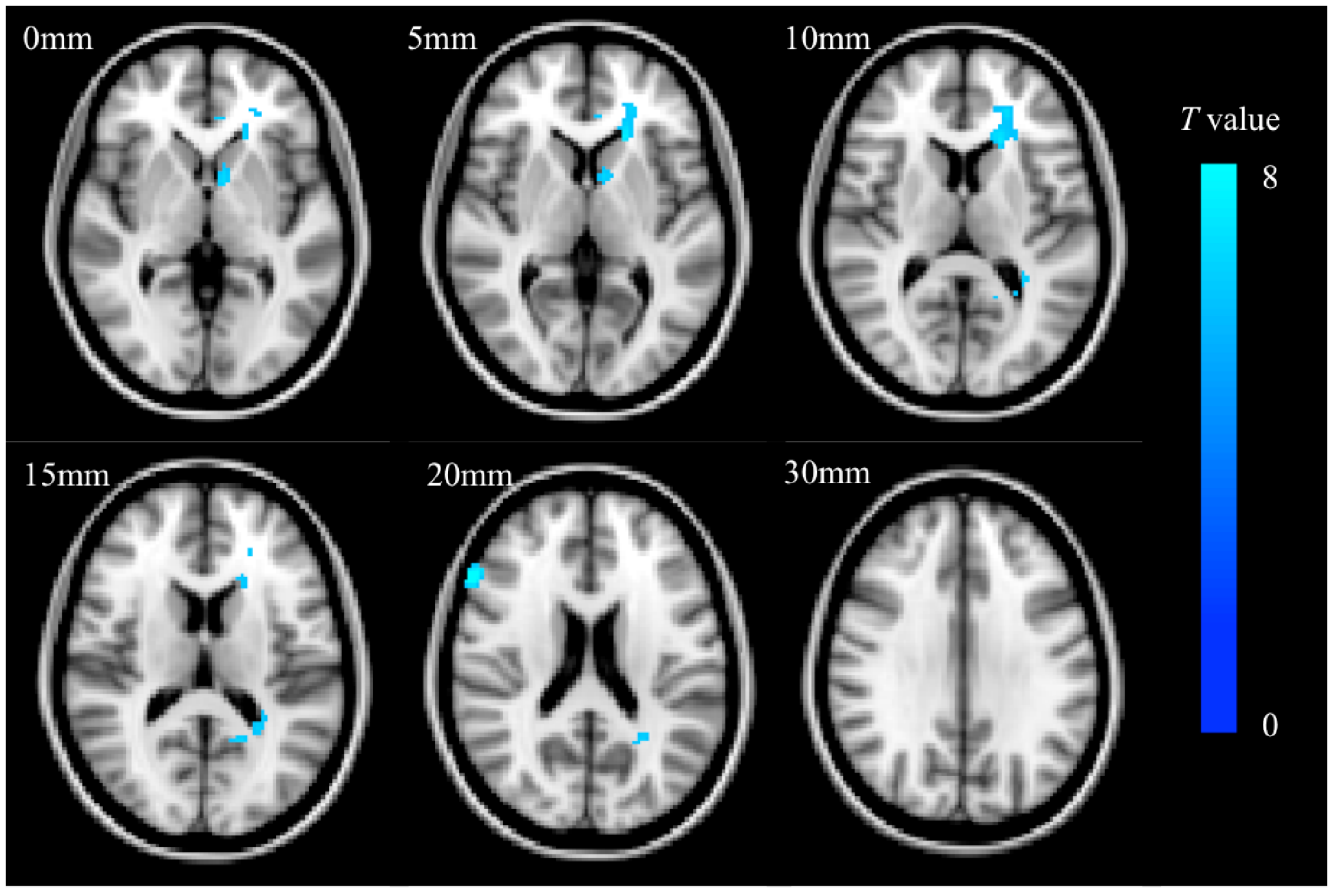
Overlap of standard-space brain regions with significantly lower FA in patients with post-stroke apathy. Colors map the *t*-values. Slice thickness  = 2 mm, *L:* left; *R*: right.

After controlling for age, education level, HAMD scores and MMSE scores, significantly lower FA was detected in four clusters with peak voxels in the genu (X = −16, Y = 30, Z = 8), and splenium (−22, 30, 10) of the corpus callosum left anterior corona radiata, and white matter in right inferior frontal gyrus (52, 24, 18) (Fig. 1, FWE corrected, *P*<0.05, *k*>50).

## Discussion

We found that depression and cognitive decline were associated with post-stroke apathy, which was consistent with previous studies [16]–[18]. However, it should be noted that some patients with moderate to severe dementia do not show apathy [19], and a recent study reported no significant overlap between apathy and depression in a group of participants 3 months after stroke [20]. Because apathy has distinct risk factors and therapeutic strategies [2], it may have a distinct neuroanatomical basis, and should be regarded as different from depression or cognitive decline.

In our present study, after controlling for age, education level, severity of depression and cognitive decline, significantly lower FA was detected in a cluster located in the left frontal corona radiata. These fibers primarily connect regions within the frontal lobe, the striatum, and thalamus, and are thus part of several frontal-subcortical loops [21]. Many studies have suggested that these loops are the most important structural feature related to post-stroke apathy [21], [22]. Indeed, they comprise multifunctional neural circuits that are closely related to emotion and cognitive function, yet have relatively functional and structurally independent. The primary structures that make up these loops include the frontal lobe, striatum, globus pallidus, thalamus, limbic system, and the fiber links between them [23]. It should be noted that there are three subtypes of apathy: emotional, cognitive, and behavioral [24]. The emotional subtype is related to the disorders of emotional signaling, which primarily result from injury to the orbital prefrontal cortex, inferior frontal cortex, and medial fiber link. The cognitive subtype is related to an inability to develop detailed plans required for action, and results primarily form injury to the dorsolateral prefrontal cortex and its fiber link. The behavioral subtype is related to a decrease in autonomous behavior compared with behavior capacity, and results primarily from damage to the medial globus pallidus the limbic system, and related brain regions [25]. Given that the left frontal corona radiata primarily connect the prefrontal cortex to the striatum and thalamus, and constitute a major part of several frontal-subcortical loops, our findings here support our hypothesis; damage to the white matter fibers that make up the frontal-subcortical loops, results in impaired emotional and cognitive functions, and thus apathy.

We also found that the FA values for the genu and the splenium of the corpus callosum were significantly lower in the apathy group than in the non-apathy group. Infarction of the corpus callosum has been linked to apathy [25], which is not surprising given that in humans it has the largest number brain fibers that connect cortical regions as well as subcortical brain structures, such as the basal ganglia across hemispheres [26]. Studies have shown that the genu of the corpus callosum includes the crossing prefrontal fibers [27]. Thus we speculate that damage to fibers in the genu of the corpus callosum (as evidenced by lower FA values) disrupts critical prefrontal connections and results in apathy. The splenium of the corpus callosum connects the occipital and temporal cortices, and when these connections are disrupted, apathy may arise because of dysfunctions in integrating multisensory information, manipulating stimuli in working memory, and re-orienting attention to relevant information [28]. Our results are in line with a study indicating that FA values of the genu and the splenium of the corpus callosum were negatively correlated with the degree of apathy observed in a group of patients with Alzheimer’s disease [26].

Our data also indicate a relationship between subcortical white matter in the right inferior frontal gyrus and post-stroke apathy. Given that the right inferior frontal gyrus is associated with several particular forms of executive control, such as response inhibition and attentional switching [29], we propose that disconnection of this brain region could lead to symptoms of apathy, due to executive dysfunction.

The present study had some limitations. First, although we noted lesion location in 16 brain regions, we did not draw the lesion maps and calculate the volume of each lesion. This might be a confounding factor and should be, taken into consideration in future studies. Second, owing to the small number of cases, studying each subtype of apathy in detail was not possible. Future studies will use larger sample sizes so that each subtype of apathy can be studied separately and more accurate conclusions can be drawn.

In conclusion, damage to the white matter integrity of the left anterior corona radiata, genu and splenium of the corpus callosum and subcortical white matter in the right inferior frontal gyrus are likely good predictors of apathy development after ischemic stroke.
